# Dynamic cumulative activity of transcription factors as a mechanism of quantitative gene regulation

**DOI:** 10.1186/gb-2007-8-9-r181

**Published:** 2007-09-04

**Authors:** Feng He, Jan Buer, An-Ping Zeng, Rudi Balling

**Affiliations:** 1 Biological Systems Analysis Group, HZI- Helmholtz Centre for Infection Research, Inhoffenstrasse, D-38124 Braunschweig, Germany; 2 Mucosal Immunity Group, HZI- Helmholtz Centre for Infection Research, Inhoffenstrasse, D-38124 Braunschweig, Germany; 3 Institute of Medical Microbiology, Hannover Medical School (MHH), D-30625 Hannover, Germany; 4 Systems Biology Group, HZI- Helmholtz Centre for Infection Research, Inhoffenstrasse, D-38124 Braunschweig, Germany; 5 Institute of Bioprocess and Biosystems Engineering, Hamburg University of Technology, Denickerstrasse, D-21073 Hamburg, Germany

## Abstract

**Background:**

The regulation of genes in multicellular organisms is generally achieved through the combinatorial activity of different transcription factors. However, the quantitative mechanisms of how a combination of transcription factors controls the expression of their target genes remain unknown.

**Results:**

By using the information on the yeast transcription network and high-resolution time-series data, the combinatorial expression profiles of regulators that best correlate with the expression of their target genes are identified. We demonstrate that a number of factors, particularly time-shifts among the different regulators as well as conversion efficiencies of transcription factor mRNAs into functional binding regulators, play a key role in the quantification of target gene expression. By quantifying and integrating these factors, we have found a highly significant correlation between the combinatorial time-series expression profile of regulators and their target gene expression in 67.1% of the 161 known yeast three-regulator motifs and in 32.9% of 544 two-regulator motifs. For network motifs involved in the cell cycle, these percentages are much higher. Furthermore, the results have been verified with a high consistency in a second independent set of time-series data. Additional support comes from the finding that a high percentage of motifs again show a significant correlation in time-series data from stress-response studies.

**Conclusion:**

Our data strongly support the concept that dynamic cumulative regulation is a major principle of quantitative transcriptional control. The proposed concept might also apply to other organisms and could be relevant for a wide range of biotechnological applications in which quantitative gene regulation plays a role.

## Abstract 2

By combining information on the yeast transcription network and high-resolution time-series data with a series of factors, support is provided for the concept that dynamic cumulative regulation is a major principle of quantitative transcriptional control.

## Background

One of the important elements of gene regulation is mediated by the binding of transcription factors to specific binding sites of promoters or other gene regulatory control regions. In eukaryotes, a combinatorial activity of specific transcription factors is generally responsible for the expression of genes in certain tissues, at specific times, or under specific environmental conditions [1-4]. Although, in a few model organisms, many of the transcription factors and their corresponding binding sites have been identified [5-11], the mechanisms of the transduction of combinatorial transcription factor activity into specific quantitative target gene expression are not understood.

**Figure 1:**
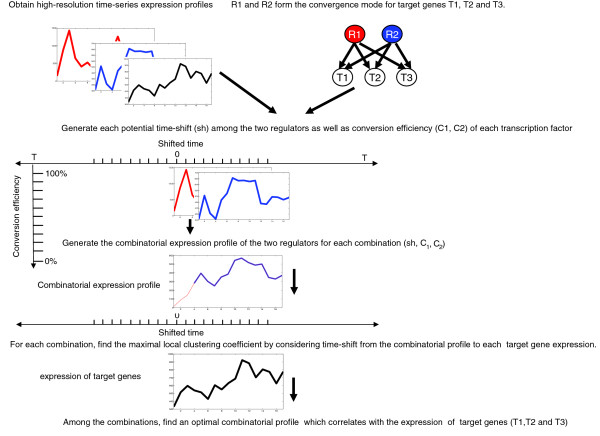
Scheme used for quantification study of combinatorial gene regulation in a convergence mode. In this example, two regulators, R1 and R2, are known to control the target genes T1, T2 and T2. Note that any two-regulator motif is not a subset of one three-regulator motif. One two-regulator motif exists if, and only if, two regulators are known to exert control on a specific target gene in the available network. At the end of this figure, no matter how statistically high or low the coefficient is, all the tests are finished for the target gene when the tests are completed in the specific convergence motif.

Eukaryotic promoters usually contain several binding motifs representing multiple-regulator-to-single-target-gene network structure motifs (regulation modes). A multiple-regulator set may control several different target genes (Figure 1), which are known as convergence network modes [12-14]. Unfortunately, limited correlation exists between the expression profile of single transcription factors and their target genes [15,16]. Attempts to strengthen this correlation by integrating time delays [17] between the expression of a regulator and its target gene have not been successful [15,16]. One of the reasons for the failure to observe a significant correlation between the expression of single transcription factors and their target genes might be that, in most cases of transcription regulation, more than one transcription factor is responsible for the activation of a target gene, leading to a combinatorial mechanism of target gene activation. Furthermore, different transcription factors not only are transcribed at different times, but also display different dynamics of translation, processing, or posttranslational modification and activation. This leads to different conversion efficiencies from the transcription of a transcription factor to fully functional regulatory activity.

In order to obtain further insight into the potential quantitative mechanisms of target gene activation, use can be made of gene expression data and knowledge of the available transcriptional gene network of yeast [18-20]. A number of recent studies have addressed the problem of gene expression. For example, Greenbaum *et al*. [21] have studied the correlation between yeast protein abundance and genome-wide mRNA expression levels and have observed only a low correlation. Similar observations have been made by Washburn *et al*. [22], Griffin *et al*. [23], and Ghaemmaghami *et al*. [24]. In these studies, comparisons of mRNA and protein levels in yeast have shown Spearman-rank correlation coefficients of only 0.45, 0.21, and 0.57, respectively. These low correlation coefficients might have resulted from measurement errors or from noise in the protein and/or mRNA levels.

An alternative explanation could be the importance of time delays between the mRNA expression of genes and the accumulation of their corresponding proteins. Le Roch *et al*. [25] have systemically compared transcript and protein levels in *Plasmodium falciparum*. In their study, strong time delays between mRNA and protein accumulation have been found, indicating the importance of this factor. The difference among these delays for individual genes encoding regulators, the difference among the time used for posttranslational modifications for different proteins, and other unknown differences will possibly cause a shift in the time at which the various regulators function. Therefore, we think another kind of potential time-shift exists among different transcription factors themselves, in addition to the well-studied delay from the time when transcription factors are expressed to the time when their corresponding target genes are induced or repressed [15,17,26,27]. Because the time delay is such an important component of gene regulation, detailed high resolution (short interval) time-series analyses have to be used in order to understand the quantitative dynamic behavior of biological systems.

Many steps are involved in the conversion of mRNA from a transcription factor gene into an activated, fully functional, binding regulator. The efficiency of each of these steps can be expected to vary from transcription factor to transcription factor, although the precise mechanisms are still unknown. Different transcription factors have different mRNA turnover rates [28,29]. P-bodies, for example, are involved in the degradation, storage, and transportation of mRNA and apparently also in the direct regulation of protein synthesis [30]. Furthermore, protein turnover [31,32] should also be considered. Assuming that only a fraction of the mRNA is translated into functional transcription factor proteins, we have assigned a conversion efficiency to the mRNA of each regulator in each convergence mode. Of note, this conversion efficiency is a comprehensive factor that integrates not only the translation from mRNA to protein, and/or posttranslational modifications, but also the assembling efficiencies of proteins into a regulator and the binding efficiencies of different regulators to their binding sites.

Complex biological systems often display nonlinear dynamic behavior. This is probably also the case for the activation of target genes as a result of the combinatorial activity of different transcription factors. Nonlinear systems are computationally extremely difficult to handle. However, approximations with linear system analysis can be useful. For example, Liao's group has developed a linear method [33] to infer regulator activities. Their analysis is based on available transcriptional regulatory networks and expression data. In the work presented here, we have also used a linear approach.

To dissect the mechanisms of quantitative combinatorial gene regulation, we have considered all the factors mentioned above. By assuming a combinatorial mode of transcription factor activity as the principle of gene regulation in cases in which multiple regulators are known to control one specific target gene, and by integrating two kinds of time-shifts and conversion efficiencies, we have developed a strategy to study combinatorial gene regulation. Not only have we considered the delays from the time when transcription factors are expressed to the time when their corresponding target genes are induced or repressed, but, for the first time, we have also taken into account time-shifts among the regulators themselves. The strategy (Figure 1) is based on a systematic search for an optimal combination of potential time-shifts and conversion efficiencies of the transcription factors in the specific convergence modes. This allows us to identify a combinatorial expression profile of regulators that best correlates with the expression of the target genes. Of note, we have not utilized the theoretically possible combinations of the regulators in the whole network, but only those regulators within a specific convergence mode that are known to exist from experimental data. In the available yeast genome-wide regulatory network, we have discovered that such a combinatorial transcription profile of regulators significantly correlates with the target gene in 67.1% of 161 three-regulator motifs and in 32.9% of 544 two-regulator motifs. These percentages reach even much higher levels among the network motifs involved in the cell-cycle process. To verify the results, we have employed another set of independent high-resolution time-series expression data [34]. A high consistency in results has been obtained. We have further found that a high percentage of motifs also shows a significant correlation in the other time-series datasets from studies of stress responses. Therefore, a shifted cumulative mode of gene regulation is a predominant principle in cases in which multiple regulators are known to control one specific target gene.

## Results

### Lack of significant correlation between single regulator and target gene expression

In general, one would expect a significant correlation between the expression profile of a regulator and its corresponding target gene. In our previous studies, we employed the Pearson correlation coefficient (PCC) [35], the local clustering (LC) coefficient [17], and trend correlation (TC) scores [15] systematically to assess the correlation of time-series transcription profiles [36] between individual regulators and their corresponding target genes among 6,105 transcriptional regulatory interactions. The specificity of these regulatory interactions was derived from various genetic, biochemical, and ChIP (chromatin-immunoprecipitation)-chip experiments in yeast [37] (see Materials and methods). In the LC and TC methods, the time-shift (time delay) between a regulator and its target gene and/or inverted relationships are considered. However, by integrating the results from the three methods used, TC [15], LC [17], and PCC [35], for only 231 out of the 6,105 (3.8%; Table 1) interactions can a significant correlation with a *P *value of 2.7E-3 between the single transcription factor and the target gene be found.

**Table 1:** Effect of different factors on the quantitative expression of target genes

	Before considering multi-regulators	Two-regulator motifs			Three-regulator motifs	
Possible time delays from regulator(s) to target genes	+	+	+	+	+	+
Conversion efficiency (non-negative)		+	+	+	+	+
Possible opposite regulation between regulators			+	+	+	+
Possible time delays among regulators				+		+
Number of significantly correlated motifs (interactions)	231	35	48	179	75	272
Number of motifs (interactions)	6,105	544	544	544	161	161
Percentage	3.78%	6.43%	8.82%	**32.9%**	21.7%	**67.1%**

Plus signs indicate that the corresponding factor is considered in the corresponding case.

### Significant correlation found through shifted cumulative regulation

We postulate that this lack of correlation might be a result of the regulation of individual target genes through the combinatorial activity of several regulators. We have addressed this problem by analyzing the time-series dataset of Cho *et al*. [36]. In their work, 745 two-regulator-to-one-target-gene motifs and 331 three-regulator motifs are represented based on the known regulatory network of yeast [20]. A two- or three-regulator set may control several different target genes in a specific convergence mode. Assuming that the time-shifts and the conversion efficiencies of transcription factors acting within a specific convergence mode regulating different target genes are similar, we constrain the time-shifts and the conversion efficiencies to the identical value in a given convergence mode. The time-shift here represents the shift between the time when the mRNA of a given regulator is expressed and the time when this transcription factor begins to regulate its target gene. Therefore, we have constrained the time-shifts among the two or three regulators to the same value across different target genes in a given convergence mode. We have only chosen convergence modes in which the same regulator set has more than one target gene (see Materials and methods). Hence, 544 out of the 745 two-regulator and 161 out of the 331 three-regulator motifs are included in this work.

In all cases to find optimal correlations, we have also integrated the well-known delay from the time when the regulators are expressed to the time when their target genes are expressed. However, we have not constrained the time when the target genes are expressed to be the same among different target genes in a given convergence mode. We have then included individual conversion efficiencies, limited to the non-negative range, in which both regulators simultaneously and cumulatively control the target gene, but without opposite activity between the two regulators. We have systematically tested the effect of all possible conversion efficiencies of individual regulators (non-negative) and of all possible time delays between the regulators and their target genes on the expression profiles of the regulators. These individual time-series profiles of the two regulators in the convergence mode have then been combined into a synthetic combinatorial time-series profile in an attempt to identify the combinatorial expression profile that best correlates with the expression of the target genes (Figure 1).

Using this approach, we have been able to obtain a significant (LC > 13, corresponding to *P *< 2.7E-3 between expression profiles of two genes (see Materials and methods)) correlation between the combinatorial profiles of two regulators and the profile of their target gene in 35 two-regulator motifs. This corresponds to 6.43% (Table 1) of all the known two-regulator motifs.

We have then taken into account a potential opposite regulation between two regulators, that is, by combining negative regulation into the sign of the conversion efficiency of transcription factors (see Materials and methods). This results in the detection of a significant correlation in additional (48 of 544 (8.82%)) two-regulator motifs, indicating the existence of opposite regulation.

However, 48/544 still represents only a small fraction of the gene regulatory motifs analyzed and indicates that other crucial factors might need to be taken into consideration. So far, the relative time-shifts among individual regulators have been neglected. Consequently, we have also considered this type of time-shift. Surprisingly, the number of gene regulatory structural motifs in which the combinatorial expression profile is now significantly correlated with a target gene sharply increases from 48 to 179 of 544 (Additional data file 1). The substantial improvement from 8.82% to 32.9% (Table 1) with regard to finding a significant correlation between the combinatorial expression profile and a target gene indicates that the time-shift among regulators is highly important.

To evaluate whether the shifted cumulative regulation as demonstrated above is a general rule for the regulation of several-regulator motifs, we have subsequently extended the same strategy from two-regulator to three-regulator structural motifs. Without consideration of a time-shift among three regulators, the combinatorial expression profiles of only 35 out of 161 three-regulator motifs are significantly correlated with the expression profile of the target gene. However, when we include a time-shift among the regulators, an additional significant increase in this correlation is observed (108 of 161 (67.1%)). Details of results are provided in Additional data file 2.

### Significant difference between results for the original and randomly generated expression data and between results for the original network and randomly generated networks

**Figure 2:**
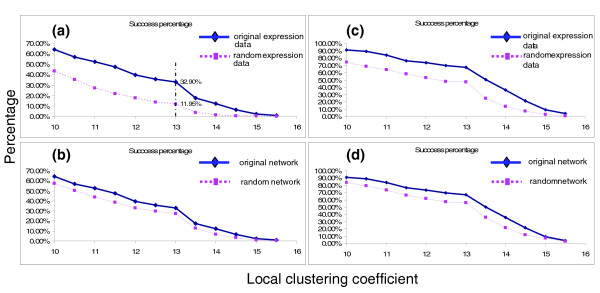
A high percentage of motifs showing a significant correlation in the real data; significantly different distribution of success percentages between real and random expression data (network). **(a) **Significantly different distribution of success percentages at different thresholds in the two-regulator motifs between the studied real expression data and randomized (shuffled between different time points) data. **(b) **Significantly different distribution of success percentages at different thresholds in the two-regulator motifs between the studied real network and random networks. **(c) **Significantly different distribution of success percentages in the three-regulator motifs between the original expression data and the randomized data. **(d) **Significantly different distribution of success percentages in the three-regulator motifs between the studied real network and random networks.

To determine whether the distribution of success percentages at different thresholds is different for the original data [36] and random data, we calculated the correlation scores of the two- and three-regulator motifs on randomly shuffled expression data and subsequently performed both paired Student's *t*-test and Wilcoxon matched-pairs signed-ranks test. We found that the success percentage (Figure 2a,c) at each threshold in the original expression data is significantly higher than that in the random data. The paired Student's *t*-test rejects the null hypothesis that the mean of success percentages at different thresholds in the original expression data is less than or equal to that in the randomly shuffled expression data for the two-regulator motifs and the three-regulator motifs (*P *= 3.35E-5 and 9.4E-7, respectively). Because we do not know whether the distributional assumption of normal-theory-based *t*-tests is satisfied in the distribution of the success percentage, we applied the Wilcoxon matched-pairs signed-ranks test (*P *≤ 4.88E-4 for both two- and three-regulator motifs). The results (Figure 2a,c) show that only about 11.9% of two-regulator motifs exhibit a significant correlation (LC ≥ 13) in the random expression data compared with 32.9% in the original expression data. The false discovery rate (FDR; see Materials and methods) is only 0.168. This is acceptable because only about 16 out of 100 motifs, in which a significant correlation can be found between the combinatorial expression of regulators and target gene expression, would be false. In the three-regulator motifs, the success percentage at threshold 13 is also lower in the randomly produced data compared with the original data. In this case the FDR is as low as 0.124.

To obtain more stringent statistical results, we have also generated random networks by randomly choosing genes as regulators and target genes. The random networks are generated by keeping the same structure for each convergence mode and keeping the expression data intact. Keeping the same structure of the convergence modes is to make sure the random networks are comparable with the real network. In this way, the statistical results are more reliable since we need to constrain the time-shift and the conversion efficiency of the same regulator to the same value for different target genes in the same convergence mode. Both paired Student's *t*-test (*P *= 6.81E-6 and *P *= 5.15E-6 for two- and three-regulator motifs, respectively) and Wilcoxon matched-pair signed-ranks test (*P *≤ 4.88E-4 for both two- and three-regulator motifs) show a significant difference between the success percentages of the original network and random networks. We have also found that the success percentage at each threshold in the original network is higher than that in random networks (Figure 2b,d). If we compare the original network with random networks, for two- and three-regulator motifs the FDR is 0.388 and 0.390, respectively, a high value when compared with criteria of traditional *P *values but acceptable for FDR. A relatively higher FDR obtained from random networks may indicate the incompleteness of the real transcription regulatory network.

Therefore, we consider that the results are significant and meaningful in real biological data. In short, the results would have been difficult to obtain haphazardly.

### Investigation of network structural motifs involved in the cell cycle

Since time control is so important in combinatorial gene regulation, if the principle of shifted cumulative regulation exists, then this mode should be more enriched in the biological processes in which time control is more essential. We thus checked the cell-cycle process to determine if this is the case. Among all of the 544 two-regulator motifs known, the target genes of 60 have been previously assigned to certain specific phases of the cell cycle of yeast [36] and/or annotated as being related to cell-cycle processes in the Gene Ontology database [38] (Additional data files 1 and 7). We found that, for 36 of these 60 motifs (60.0%), the combinatorial profile of the two regulators is significantly correlated with the expression of the target gene. In most of these 36 motifs, at least one regulator has been assigned to certain phases of the cell cycle or annotated to the cell-cycle process. Among the 161 three-regulator motifs, the target gene of 34 motifs has been assigned to the cell cycle. Remarkably, 30 out of the 34 three-regulator motifs (88.2%) show a significant correlation between the combinatorial expression profile of the three regulators and their target gene. Consistent with this expectation, such high percentages have further strengthened the idea that shifted cumulative regulation is one of major principles in quantitative expression control.

**Figure 3:**
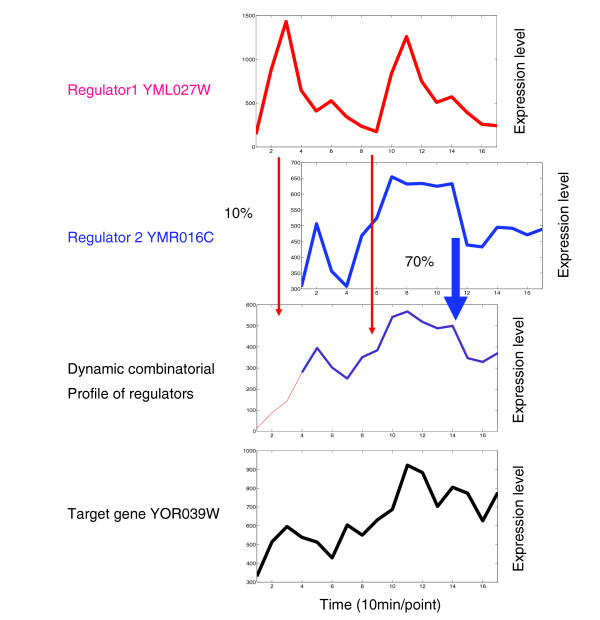
Shifted cumulative regulation. Illustration of the concept that transcription expression profiles (non-normalized) of regulator YML027W (YOX1, red line) and regulator YMR016C (SOK2, blue line) are dynamically combined. This demonstrates a significant match between the combinatorial expression profile and the expression of the target gene YOR039W (CKB2) in the studied dataset. The conversion efficiency, which indicates the ratio between the number of functional activated binding regulators and the number of available transcription factor transcripts, is presented as a percentage (10% and 70% here).

Shifted cumulative regulation can be nicely demonstrated in the following example. In yeast, the transcription factors YML027W (YOX1) and YMR016C (SOK2) have been described to regulate the transcription expression of YOR039W (CKB2) [20]. The latter is reported as a G1/S transition gene and as a G2/M transition gene [39]. YOX1 is reported to be one of the G1/S-specific transcriptional genes [37]. Cho *et al*. [36] have also observed that YOX1 belongs to the late G1 phase. We therefore expect a significant correlation between YOX1 or SOK2 and the target gene CKB2. However, using the PCC, LC, and TC methods, a significant correlation between YOX1 and CKB2 could not be detected, as indicated by the corresponding parameters (scores of 0.32 for PCC, 7.41 for LC, and sc 12 and cc 0.71, respectively, for TC). Similarly, a significant correlation cannot be detected between SOK2 and CKB2. By the mode of shifted cumulative gene regulation, these results can now be explained. As shown in Figure 3, the combinatorial profile of the two regulators (YOX1 and SOX2) correlates highly significantly (13.1, corresponding to *P *= 2.7E-3 between expression profiles of two genes) with that of the target gene (CKB2). We also show the time-shifts and the conversion coefficients of the regulators derived for the regulation of CKB2 in Figure 3. Based on our analysis, there is a delay of three time points (about half an hour) for SOK2 compared with YOX1, and only 70% and 10% of the mRNAs of SOK2 and YOX1, respectively, seem to be converted to functional activated binding regulators activating CKB2. These results strongly suggest that shifted cumulative regulation exists.

### Consistency of time-shifts and conversion efficiencies of a given regulator with different target genes

A given regulator might display some similarities in quantitatively controlling its different target genes. Therefore, we examined whether these similarities occur in our results. In our algorithm, the time when a given transcription factor begins to function is already constrained to an identically shifted time point among different target genes in the same convergence mode. Hence, the time-shifts among the two or three transcription factors are kept constant for different target genes in the same convergence mode. The algorithm itself first guarantees the consistency of time-shifts for a given regulator across different target genes within the same convergence mode. Within the entire transcription network known so far, there are a total of 78 regulators contributing to two-regulator motifs (Additional data file 1). Out of the 78 regulators, 34 regulators are involved in only one convergence mode. So, the time-shifts of these 34 regulators are completely consistent among different target genes.

Next, we asked whether the time-shifts of a given regulator in different convergence modes are concordant among different target genes since one of the two regulators in a convergence mode might also be a regulator in other convergence modes. Because of computational explosion, we cannot constrain the time-shifts of a given regulator for all different target genes in the whole regulatory network to one shifted time point. Therefore, if an enriched distribution of shifted time points occurs in a short contiguous time window for a given regulator, the shifted time points of that regulator are consistent among different convergence modes.

**Figure 4:**
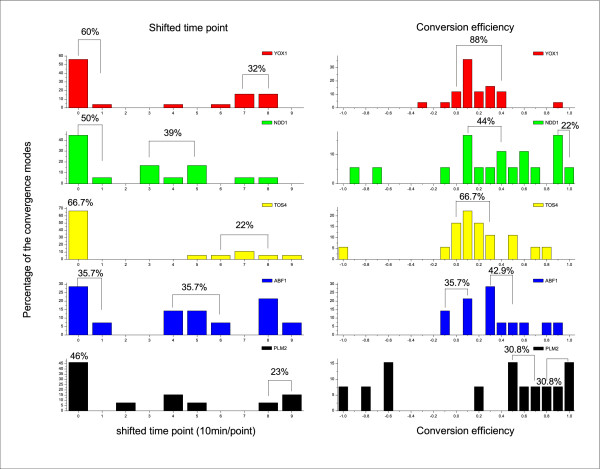
Significant consistency among different target genes in shifted time points and conversion efficiencies of the same regulator in different convergence modes. This figure shows only the top five regulators that are involved in the largest number of convergence modes. Note that one convergence mode includes more than one two- or three-regulator motif in this work. The overall percentage among all the convergence modes that the given regulator controls is indicated above the corresponding contiguous columns of shifted time points or conversion efficiency. The possibility to obtain this kind of contiguous concentrative distribution was examined by binomial distribution test. These statistics results are very significant (Additional data file 1).

Among 44 regulators that are involved in more than one convergence mode, there are 6 regulators, each of which shifts to an identical time point in different modes. Therefore, the six regulators show a perfect consistency among different target genes in the whole regulatory network in terms of shifted time point. For each of 27 out of the other 38 regulators, the shifted time points of different convergence modes mainly concentrate in one or two areas (*P *< 5E-2; Additional data file 1). Each area comprises a short (one to three time points) contiguous time window (Figure 4). For example, the regulator YML027W (YOX1) is the regulator of 25 two-regulator convergence modes (including 92 two-regulator-to-single-target motifs). In 14 out of the 25 modes, the time when the regulator YOX1 begins to function relative to the first time point is zero. In 8 out of the 25 modes, the time when YOX1 begins to control its target genes shifts to time point 8 or 9 in a concentrative pattern (Figure 4). The binomial distribution test shows that it is very difficult to obtain 8 out of the 25 modes distributing in two contiguous time points from the 10 possible time points by chance (*P *= 1.25E-2; see Materials and methods). The distribution pattern of shifted time points of a given regulator appears to be concentrative (Additional data file 1 and Figure 4). This concentrative pattern demonstrates a good consistency of the shifted time points among different convergence modes and, consequently, across different target genes in the entire transcription network analyzed.

Our algorithm also constrains the conversion efficiencies of a specific transcription factor among different target genes to an identical value in a given convergence mode. Therefore, to further assess the consistency of the conversion efficiencies of given regulators in the whole transcription network, we only need to check whether the conversion efficiencies of those regulators in different convergence modes distribute in a concentrative manner. Forty-four regulators are involved in more than one convergence mode. One regulator has the same time-shift among different convergence modes. For each of 29 out of the 43 regulators, the shifted time points in different convergence modes mainly concentrate in one or two areas (Additional data file 1). Each area comprises a short (one to five points - only one regulator distributes in five points) contiguous conversion efficiency window. For example, in 22 out of the 25 modes, the conversion efficiencies of YOX1 only distribute in the short range 0-0.4 (Figure 4). The binomial distribution test shows that 22 out of the 25 modes distribute in 5 contiguous conversion efficiencies of the 21 possible conversion efficiencies. This cannot be randomly obtained (*P *= 1.65E-14). Therefore, the conversion efficiency of a given regulator is also quite consistent among different convergence modes and, hence, consistent among different target genes in the whole available transcription regulatory network analyzed.

In addition, the analysis of variance of time-shifts and conversion efficiencies for each regulator across different convergence modes has also shown a similar outcome as the above analysis of the short contiguous distribution. The variance of time-shifts (conversion efficiencies) of a given regulator is measured by the standard deviation of time-shifts (conversion efficiencies) among different convergence modes that the given regulator controls. We take the standard deviation of time-shifts (conversion efficiencies) of all the regulators across all the different convergence modes as background deviation. It turns out that 25 out of the 38 regulators show a smaller standard deviation of time-shifts than the background deviation (Additional data file 1). We have observed that 29 out of the 43 transcription factors have a smaller standard deviation of conversion efficiencies than the background deviation (Additional data file 1).

### Validation in another independent dataset

If, for the same multi-regulator transcriptional regulatory network motifs, the shifted cumulative regulation can also be found in another independent dataset, these results would corroborate our discoveries. For this purpose, we have utilized the high-resolution time-series yeast expression dataset of Spellman *et al*. [34]. In their data, which was also originally used for analyzing genes involved in the cell-cycle process, the same time interval (ten minutes) was employed for microarray measurements. Therefore, it represents a good opportunity for the confirmation or refutation of our results. Because of the values missing at some time points in this microarray dataset (see Materials and methods), we have been able to find only 208 common two-regulator motifs that exist in both the Cho and the Spellman time-series datasets [36]. In 59 out of the 208 two-regulator motifs, a significant correlation between the combinatorial expression of the regulators and the target gene expression has been found in the Cho time-series dataset. Of the 208 two-regulator motifs, 67 show a significant correlation in the Spellman dataset. Among them, 21 two-regulator motifs show a significant correlation in both the Spellman dataset and the Cho time-series dataset (Additional data file 3). For the three-regulator motifs, the intersection of the motifs between the Spellman data and the Cho data is only 32 (Additional data file 4). Up to 25 out of the 32 motifs show a significant correlation in the Cho data and 20 are significant in the Spellman data. The overlapping number of significant correlated motifs in both the Spellman and the Cho data is 16.

We then examined whether these overlapping numbers of 21 and 16 could be obtained by chance. If we assume there are only 59 motifs showing a significant correlation in the whole population of 208 two-regulator motifs, the possibility to obtain 21 or more significant motifs by randomly taking 67 motifs is 0.31 (hypergeometric test). The possibility to obtain 16 or more significant three-regulator motifs can also be calculated by hypergeometric test (*P *< 0.53). These possibilities alone are not significant in terms of the chance to obtain these overlapping numbers. However, these tests alone cannot justify whether these overlapping significant motifs could be easily obtained by chance. We need to further evaluate whether the other aspects of these common significant motifs are consistent between the two experiments. One could expect that sometimes these overlapping numbers could be obtained by chance, although one could not also expect that the accordance of the time-shift and the conversion efficiency between the two experiments could be obtained in the common significant motifs by chance. Note that the consistency of the time-shift and the conversion efficiency between the two experiments is independent of the consistency in significance of correlated scores of the motifs.

We therefore examined whether the time-shift and the conversion efficiency are significantly consistent between the two experiments. Among the 42 regulators of the common significant two-regulator motifs, the difference in the time-shifts between the two experiments for 25 regulators is less than or equal to 2 time points (Additional data file 3). The binomial distribution test shows that the possibility to have a difference less than or equal to 2 time points in a concentrative way for 25 regulators among a total of 42 regulators is 3.35E-6. Therefore, even if one could obtain these 21 common significant motifs by chance, it is still very difficult to obtain a difference in time-shift less than or equal to 2 time points for 25 regulators between 2 experiments by chance. Furthermore, we tested whether the consistency of the conversion efficiency could be obtained by chance. Among the 42 regulators of the common significant motifs, the difference in the conversion efficiency between the 2 experiments for 19 regulators is less than or equal to 0.3. The binomial distribution test was used to examine the possibility that the difference between the two experiments in the conversion efficiency concentrates in the short contiguous window less than or equal to 0.3 for 19 regulators among 42 regulators (*P *< 2.52E-7). The total *P *value to obtain this number of overlapping significant motifs with a significant consistency in both time-shift and conversion efficiency is 2.61E-13.

Analogously, the possibilities to have consistency in both time-shift and conversion efficiency for the three-regulator motifs are significant (*P *< 4.3E-3 and *P *< 1.8E-4, respectively). Taken together, even if one could obtain the overlapping numbers of significant motifs by chance, it is also very difficult to obtain a highly significant consistency between the two experiments in both time-shift and conversion efficiency by chance.

Because the Spellman dataset is an independent dataset, the highly consistent results have confirmed the findings obtained for the Cho dataset. Additionally, compared with data in the Cho dataset, similar results were obtained, that is, in 72 of 219 (32.9%) two-regulator motifs and 25 out of 38 (65.8%) three-regulator motifs, a significant correlation can be found in the Spellman dataset; these similarities indicate that shifted cumulative regulation is a major principle for multi-regulator transcriptional network structure motifs.

In short, for both two- and three-regulator convergence motifs, it is very difficult to obtain this kind of observed overlap between the Spellman and Cho datasets by chance. These results have excluded the risk of overfitting.

### Shifted cumulative regulation is also dominant in feed-forward loops

The feed-forward loop (FFL) has been found to be over-represented in various biological systems [16,40-43]. A FFL is composed of three nodes. A transcription factor regulates a second transcription factor, and both regulators also regulate the target gene. Therefore, a FFL has two parallel regulation paths: a direct path from the first regulator to the target gene and an indirect path that goes through the second regulator. Because of the structural characteristics of FFLs, a two-regulator-to-single-target-gene structure might actually be a FFL. Since the first regulator can directly and indirectly regulate the target gene, this additional functional characteristic might affect the quantitative regulation mechanism of the target gene. Similarly, an FFL may also be involved in a three-regulator-to-single-target structure.

Hence, we have evaluated whether there is a significant difference between the FFL and non-FFL groups in terms of the frequency of shifted cumulative regulation. Among all of the 544 two-regulator motifs from the Cho dataset, 73 motifs are also FFLs (Additional data file 1). Of these 73 motifs, 27 (37.0%) show a significant correlation between the combinatorial expression profile of the regulators and the expression of the target gene. Among the 471 non-FFL two-regulator motifs, a significant correlation is found in 152 motifs. The Yates chi-square test has been used to determine the difference between the success frequencies of the FFL and non-FFL groups. The results (chi-square = 0.44, df = 1, *P *= 0.507) show that there is no significant difference in the two-regulator motifs in the Cho dataset. FFLs are also involved in 29 three-regulator motifs. A high percentage (21 out of 29 (72.4%); Additional data file 2) shows a significant correlation between the combinatorial expression of the regulators and the target gene expression. In 87 out of the 132 non-FFL three-regulator motifs (65.9%), a significant correlation has also been detected in the Cho dataset. The difference (Fisher's exact test, *P *= 0.329; see Materials and methods; Additional data file 3) between the FFL and non-FFL groups in the three-regulator motifs is also not significant in the Cho dataset. Similarly, there is no significant difference between the FFL and non-FFL groups for the two-regulator motifs (Fisher's exact test, *P *= 0.558) and three-regulator motifs (Fisher's exact test, *P *= 0.429; Additional data file 4) from the Spellman dataset. Thus, even in the FFLs, shifted cumulative regulation is also a major principle. Although the first transcription factor can regulate the target gene twice, by a direct path and an indirect path, only the second regulator directly regulates the expression of the target gene in the indirect path. Therefore, the first regulator and the second regulator directly regulate the target gene only once *per se*. This is the reason that the frequency of shifted cumulative regulation is similar in the groups of FFLs and non-FFLs.

### Shifted cumulative regulation is also applicable to stress-response conditions

To examine whether the principle of shifted cumulative regulation only prevails in the synchronized yeast cell cultures, we next performed a similar analysis under other conditions, such as stress responses. Because the high-resolution time-series expression data with equal sampling interval were required for this analysis, we chose only two conditions from the available data. The first one was originally used for studying the transcriptional response of steady-state yeast cultures with a low-level glucose pulse perturbation [44]. The second one was utilized for an analysis of expression in the response of yeast cells to constant 0.32 mM hydrogen peroxide (H_2_O_2_) stress [45].

Under the condition of low-level glucose pulse perturbation, 557 two-regulator motifs are included on the available regulatory network (see Materials and methods). If we choose the same *P *value cutoff (2.7E-3; see Materials and methods), 141 out of the 557 two-regulator motifs (25.3%; Additional data file 5) show a significant correlation between the combinatorial expression of the regulators and the target gene expression. The data obtained under H_2_O_2 _stress include 453 two-regulator motifs. Among them, a significant correlation can be detected in 114 two-regulator motifs (25.2%; Additional data file 6). These success percentages are higher in three-regulator motifs under both conditions (45.2% of 168 motifs and 47.5% of 120 motifs for glucose pulse perturbation and H_2_O_2 _stress, respectively; Additional data files 5 and 6). These success percentages are relatively lower than those in the data used to study cell-cycle regulation. However, percentages of approximately 45% and 25% are still considered to be high at the systems level. Consequently, we can conclude that shifted cumulative regulation is also applicable to other conditions, rather than only being constrained to the synchronized yeast cell cultures, which were originally used to study cell-cycle regulation.

## Discussion

Major efforts are currently directed toward the identification of the components of biological systems. These include the sequencing of whole genomes and the analysis of genome-wide expression profiles of transcripts or proteins in specific physiological or pathophysiological states. However, mere knowledge of the components is not sufficient to reveal the complexity of biological systems. We also need to understand the dynamics of the interactions between the individual components.

In the work presented here, we have used genome-wide high-resolution (short interval) time-series expression data from yeast [36] in order to understand some of the basic principles that underlie quantitative gene regulation. The relationship between transcription factors and their target genes can be analyzed by a correlation analysis between the regulator(s) and the induction of the corresponding target gene(s). Unfortunately, the attainment of significant correlations between one single transcription factor and a specific target gene has not been straightforward.

A number of groups have tried to carry out genome-wide correlation analyses, for example, by using the PCC [35] to identify relationships between regulators and target genes [15-17]. In these studies and also in the present work, only a small percentage of significant relationships has been found between the expression of single transcription factors and their target genes, even when time-shifts [15-17] are included in the analysis (Table 1).

We have hypothesized that this might be attributable to the finding that most genes are regulated through the combinatorial activity of more than one transcription factor. We have also considered potential differences in the conversion efficiencies between the transcription of individual regulators and their functional activity. Because many factors contribute to the conversion of a transcription factor transcript into a functional binding regulator, a coefficient representing this conversion efficiency has been integrated into our analysis. Such a conversion efficiency factor needs to be looked at as a comprehensive parameter, integrating factors such as differences in the translation efficiency from mRNA to protein, in the assembly efficiency from protein to regulator, in posttranslational activation (inhibition), and in the binding efficiency of the regulators to their binding motifs. We derive these conversion efficiencies by testing all possible conversion efficiencies of the transcription factors of the convergence mode in order to find the specific combination of conversion efficiencies to form a combinatorial expression profile of the transcription factors that best correlates with the expression of the target gene. A specific regulator can display different conversion efficiencies dependent on the specific convergence modes.

As shown in Table 1, we obtained a significant correlation between regulators and target genes of yeast by considering a multi-regulator mode of gene regulation and by integrating a conversion efficiency factor for the various regulators. After combining the time-series profiles of the two individual regulators into the combinatorial time-series profiles, the combinatorial profiles of two regulators is significantly correlated in 6.4% of cases with the induction of their target gene compared with only 3.8% when single regulators are considered (Table 1). Allowing for the potential opposite regulation of the individual regulators, this percentage increases to 8.8%. For those target genes that are known to be regulated by three regulators, a significant correlation between the expression of regulators and target genes is found in as many as 21.7% of cases.

The influence of time delays between regulators and target genes is a well-known phenomenon and is considered in all our calculations. In addition to time delays between regulators and target genes, time delays among regulators themselves might also be important. When we incorporate the influence of this second type of time delay, a further significant increase from 8.8% to 32.9% in identifying a significant correlation of regulators and target genes is obtained for the two-regulator motifs. For the three-regulator motifs, this percentage even increases to 67.1%. This dramatic increase demonstrates the extreme importance of the time-shift when different transcription factors begin to regulate the transcriptional expression of target genes. The time-shift among regulators is mainly attributed to the intrinsic asynchronous characteristics of activation/inhibition of genes or proteins. Possibly, these built-in characteristics of genes or proteins are also required for the delicate dynamic regulation of the genes or proteins. In fact, exquisite quantitative expression, rather than simple on-off expression, is also reported to be biologically functionally required in a recent study by Wan and Flavell [46]. Therefore, our proposed shifted cumulative regulation mechanism might possibly have evolved to meet the complicated spatial and temporal dynamics of gene function in organisms.

The proposed shifted cumulative model here assumes that the regulators control the target expression independently. However, in some cases, the regulators may form a heterodimer and then act on the promoter or other regulatory regions of the target gene, for example, SBF (SWI4-SWI6) and MBF (MBP1-SWI6) [47,48]. If the regulators are dependent, the whole complex of regulators is only active if both proteins are present. In such a model, the total activity should correspond to the minimum concentration of MBP1 and SWI6 (or SWI4 and SWI6, respectively). Hence, the cumulative (additive) mode that is assumed in equation 2 (see Materials and methods) is not fulfilled in such cases.

Given regulators might exhibit some similarities in quantitatively regulating the transcription expression of their different target genes. We determined the consistency of the time-shifts and the conversion efficiencies of given regulators among different target genes in the same convergence modes by integrating a direct constraint in our algorithm. Our results also demonstrate that the time-shifts and the conversion efficiencies of given regulators are significantly consistent among their different target genes in different convergence modes.

As discussed above, conversion efficiency is a comprehensive parameter, integrating factors such as differences in the translation efficiencies from mRNA to protein, in the assembly efficiencies from protein to regulator, in posttranslational activation (inhibition), and in the binding efficiencies of the regulators to their binding motifs. In general, for a given regulator, the translation efficiency from mRNA to protein is assumed to be independent of its target genes. However, other factors, such as assembly efficiencies from protein to regulator and posttranslational processes, may still be dissimilar because several different signaling pathways or mechanisms might possibly be involved in those processes for a given regulator. For example, MYC responds differently to different inputs from other factors and/or signals [49]. Another well-known example is represented by transcription factors activated by mitogen-activated protein kinase (MAPK) cascades [50]. The binding efficiencies of a given regulator for different target genes might also be distinct. For instance, the particular arrangement of sites at target promoters is also an important influence on MYC activity; docking MYC at different distances from the transcription start site modulates its activity [51]. At different promoters, MYC may act through different mechanisms and at different stages of the transcription cycle [52]. The detailed mechanisms that lead to a specific conversion efficiency of individual transcription factors are generally not known. A given regulator can be activated through different pathways and, subsequently, induce different target genes. This is supported by a recent report [53] that shows that the strengths of a given regulator on different target genes are different because of the different binding positions in the gene promoter, when considering expression data under independent conditions. Therefore, it is not surprising to observe some divergences in the conversion efficiencies of given regulators for different target genes.

The time-shift among the two or three regulators is the same for their different target genes in the same convergence mode. It is also reasonable that different target genes in different convergence modes show some differences in the time-shifts when a given regulator begins to function to control its target genes relative to the time when the mRNA of that regulator is expressed. One reason for this could be that the regulator may activate its different target genes at different times [49,54,55]. Since a given regulator may be activated through different mechanisms or pathways and subsequently induce different target genes even within the same cell [49,50,56], some temporal difference might exist among the different pathways. We cannot exclude that such different mechanisms or different pathways exist, for example, in synchronized yeast cell cultures. Hence, it is possible that differences in time-shifts among given regulators occur in different motifs.

The lower percentage of two-regulatory motifs compared with three-regulatory motifs showing a significant correlation between combinatorial expression profiles of regulators and the expression of target genes might be the result of incomplete knowledge concerning the structure of the underlying gene regulatory network. Among both two-regulator- and three-regulator motifs, some parts cannot be well explained by our proposed cumulative regulation modes. One alternative possibility is that the utilization of promoter regulatory modes are condition- or environment-dependent [57]. Another explanation is that, although the microarray quality control project has shown inter- and intra-platform reproducibility of gene expression measurements [58], the expression data used here can be affected by many artifacts and/or experimental errors [59,60]. The cell-cycle expression data also suffer from the synchronization loss problem. To address this problem, Bar-Joseph *et al*. [61] once proposed an approach to deconvolve cell-cycle expression data by utilizing some complementary information, such as fluorescence-activated cell sorting analysis and budding index. The deconvolved values can be used to improve the synchronization loss problem. However, we prefer to use observed values here, because deconvolved values are only applicable to genes that are involved in the cell cycle. In this work, we have also been interested in other genes that are involved in other pathways but that might also be regulated in the synchronized yeast cell cultures, which were originally used to study cell-cycle regulation. In addition, de Lichtenberg *et al*. [62] once proposed an approach in which the timescales are first transformed from minutes to percentages of the cell cycle in different experiments in order to obtain one integrated global peak time. This method was demonstrated to be useful to identify periodically expressed cell-cycle genes. However, in this work, we want to know whether the mechanism of shifted cumulative regulation is a general principle of quantitative gene regulation under different conditions and/or in different experiments. We are interested in not only the peak expressions but also all the other quantitative expression values over time. Because of the exponential increase in computer time needed (for the number of combinations in two- or three-regulator motifs, see Additional data file 8), we have not analyzed motifs with more than three regulators.

Stringent time control is an intrinsic property of the cell-cycle process. Therefore, the mechanism of shifted cumulative transcription factor activity might be a particularly prominent feature of regulatory motifs in the target genes involved in the cell cycle. Remarkably, out of 60 two-regulator motifs, in which at least the target gene has been assigned to certain phases of the cell cycle [36] and/or annotated to be related to cell-cycle process [38], our analysis reveals that 60% of these motifs show a significant correlation between the combinatorial expression profile of regulators and their target gene expression. For three-regulator motifs, the target gene of 34 motifs has been assigned to and/or annotated to the cell cycle. In 88.2% of the 34 motifs, a significant correlation can be detected between the combinatorial profile of three regulators and the expression of their target genes.

To validate the principle of shifted cumulative regulation in multi-regulator transcriptional regulatory network motifs, we have utilized another independent set of high-resolution time-series expression data [34]. We have found a considerable intersection of the two- or three-regulator motifs showing a significant correlation in both the Spellman and the Cho data. There is a significant consistency between the two datasets with regard to time-shifts and conversion efficiencies for given regulators. But some differences in the time-shifts and the conversion efficiencies of given regulators between the two datasets might originate from the innate differences between the two experiments. Cho *et al*. [36] published their data using the temperature-sensitive mutant strain cdc28-13 but the Spellman dataset is based on the cdc15 temperature-sensitive mutant. The cdc28-13 mutant can arrest the yeast cell in late G1 phase, whereas the cdc15 mutant is arrested in late G2 phase.

We have further found that shifted cumulative regulation is also dominant in FFLs. Many two-regulator motifs are actually FFLs. FFLs are also involved in some three-regulator motifs. Because, in FFLs, the first regulator can directly and indirectly regulate the target gene, this additional function characteristic may affect the quantitative regulation mechanism of the target gene. However, the results show that the frequencies of identifying a significant correlation in FFL and non-FFL groups are not significantly different. This is readily understandable because, although the first regulator can directly and indirectly regulate the target gene twice, the indirect pathway functions via the second regulator. Eventually, the first regulator and the second regulator directly regulate the target only once. Therefore, no matter what form the middle processes take, the final results of FFL regulation are similar to that of normal convergence mode regulation.

We want to note that the shifted cumulative regulation mode is also applicable in other conditions and not only in synchronized yeast cell cultures. The results demonstrate that a considerable fraction of two- and three-regulator motifs also shows a significant correlation under stress conditions, such as H_2_O_2 _stress and glucose pulse perturbation. The success percentages in the two studies under stress conditions are relatively lower than those in the data used to study the cell cycle. This may stem from the fact that some of the transcription factors are not regulated at the transcriptional level in response to stress.

## Conclusion

In this work, we provide a strategy to dissect the basic regulatory principles of multi-regulator transcriptional regulatory networks. Our results point to a dynamic quantitative linear combinatorial model of gene regulation. We confirm the results with high consistency in two independent high-resolution time-series datasets. In addition, a significant difference that exists between results obtained for real and randomly generated data strengthens the biological relevance of this observation. The success percentages of finding a significant correlation between the combinatorial expression profiles of regulators and their target gene expression among the studied motifs are even higher among regulatory network motifs involved in the cell-cycle process. We further demonstrate that the success frequencies of the shifted cumulative regulation mode are similar between the FFL and non-FFL groups. We also found that the shifted cumulative regulation mode is dominant under other stress conditions, rather than being restricted to datasets from cell-cycle studies. Taken together, our data strongly indicate that shifted cumulative regulation is a predominant principle underlying the quantitative gene regulatory mechanism of multi-regulator transcriptional regulatory network motifs. The model presented here provides evidence, for the first time, regarding the mechanism of the quantitative regulation of target genes by multiple transcription factors.

In order to understand the mechanism of gene regulation, therefore, not only is it important to follow the expression profile of single transcription factors over time, but the expression of quantitative combinations of regulators over time should also be considered. This can be achieved only through high-resolution time-series measurements. We believe that the proposed strategy can also be utilized for understanding quantitative gene regulation in other organisms.

Our strategy allows us to estimate the relative time when each of the different regulators in a specific motif begins to function. We can also estimate how much mRNA transcribed by a transcription factor gene is translated into a fully functional binding regulator. This strategy will become even more powerful with future improvements in our knowledge concerning the components of regulatory network structure and expression measurement technology. The proposed concept might be relevant for a wide range of biotechnological and biomedical applications in which quantitative gene regulation plays a role. It also provides a new perspective for experimental biologists to reveal the real quantitative multi-dimensional mechanisms of complex regulatory systems.

## Materials and methods

### Quantification of shifted cumulative regulation of gene expression: principle of the approach

To study the basic quantitative principles of gene regulation, we carried out a correlation analysis between combinatorial profiles of regulators and their target genes within regulatory network structure modes in which multiple regulators are known to control a specific target gene. For this purpose, we propose a shifted cumulative mode (Figure 1) of gene regulation that takes into account the following factors: the cumulative combinatorial activity of transcription factors in network structure motifs; potential time delays (time-shift) 'among' regulators; potential time delays 'from' regulators 'to' their target genes; and conversion efficiencies of transcription factor mRNAs into functional binding regulators. The nature of positive or negative regulation is combined into the sign of the conversion efficiency of the transcription factor. By systematically testing all possible conversion efficiencies and shifting all potential time delays among regulators within the specific convergence mode and time delays from regulators to their target genes, a combination of two kinds of time-shifts and conversion efficiencies can be identified (Figure 1). Through this combination, a combinatorial expression profile of the regulators that best correlates with the expression of the target genes can be obtained.

### Conversion efficiency and time delay among regulators

A conversion efficiency *C *for the mRNA of each regulator gene is assigned for a given convergence mode. Numerically, for each regulator *R*_*i *_in each motif, a constrained conversion efficiency *C*_*i*_(-1 ≤ *C*_*i *_≤ 1) is chosen. This is based upon the assumption that the probability that all the expressed mRNA of one regulator gene can be finally converted into the fully activated binding regulator is low, as discussed above. A negative *C*_*i *_value for a regulator means it has a regulation function (activation or suppression) opposite to that (suppression or activation) of a regulator with a positive value.

The approach is implemented in computer programs (programs are available on request) for quantifying the shifted cumulative regulation of genes in large-scale high-resolution time-series gene-expression profiling data. The major original aspect of our method is the combination of expression profiles of the regulators by considering time delays among regulators and conversion efficiencies of regulators in network structure motifs. This is illustrated below for a two-regulator-to-single-target-gene motif with *n *successive time points. The subscripts *i1 *and *i2 *are used to represent the regulators 1 and 2, respectively. Assuming *sh *for the relative time-shift between the two regulators and *R*_*i*,*j *_for the expression level of the regulator *i *at time point *j*, the expression level *A*_*k*,*j *_of the combinatorial profile of the two regulators at time point *j *in the given motif *k *can be calculated as follows:

If -*n *<*sh *< 0

   For *j *= 1 to *abs*(*sh*)

   *A*_*k,j *_= *abs*(*R*_*i*2,*j *_× *C*_*i*2_) (equation 1)

   Next

   For *j *= *abs*(*sh*) +1 to *n*

   *A*_*k,j *_= *abs*(*R*_*i*1,*j*-*abs*(*sh*) _× *C*_*i*1 _+ *R*_*i*2,*j *_× *C*_*i*2_) (equation 2)

   Next

End

For a positive time-shift (0 <*sh *<*n*, for example, the *sh *is positive 3 in Figure 3), the combining (assembling) process can be performed in a similar way. For the extreme situation in which the time-shift equals -*n *or *n*, that is, only one regulator actually functions to control the target gene in the measured time period, the combinatorial profile can be easily obtained by choosing one regulator; for simultaneous regulation (*sh *is zero), *A*_*k*,*j *_at each time point of the combinatorial profile can be calculated by equation 2.

If a third regulator exists, after the profiles of two regulators are combined, the profile of the third regulator will be combined with the combinatorial profile in a similar way as outlined above for the two-regulator motif. More regulators, if they exist, can be subsequently combined in a similar manner.

We want to emphasize that the time when a given regulator begins to function to control its target genes is constrained to an identically shifted time point among different target genes in a given convergence mode. Therefore, the time-shifts among the two or three regulators are kept the same for their different target genes in a specific convergence mode. The same applies to the conversion efficiency of the regulators within a given convergence mode.

To make the results more biologically reasonable, we have also constrained the maximum potential shift from the time when the mRNA of a given regulator is expressed to the time when the regulator begins to function to one cell cycle (approximately ten time points) in the data used for the cell-cycle study.

### Time delay from regulators to target genes

We then calculate the maximum local alignment (match), called the LC coefficient [17], between the combinatorial profile of the regulators and the expression profile of the target gene. In our approach, we consider the time-shift between the combinatorial regulator and the target gene. This kind of time-shift is calculated by a dynamic programming-based method [63] as detailed elsewhere [15,17]. We should point out that the time-shift 'from' the combinatorial regulator profile 'to' the target gene profile is different from that 'among' the regulators themselves.

All the possible values of *C*_*i *_from -1 to 1 with a step length of 0.1 for each regulator are repeatedly tested in the constrained network structure motif in which the target gene is controlled. There are 2*n *+ 1 possible values of time-shift (-*n *≤ *sh *≤ *n*) between two regulators. Finally, we choose the optimal local clustering coefficient to infer the potential holistic correlation between all the regulators and the target gene in the corresponding motif. Several criteria can be used to determine optimal correlation. The first one is to find the maximal mean value of the local clustering coefficients across all the target genes in a given convergence mode. The second criterion is to find the maximum number of target genes showing a significant correlation between the combinatorial expression profile and the target expression according to the given threshold in a given convergence mode. If there are several different possible combinations of time-shifts and conversion efficiencies, through which the same number of target genes showing a significant correlation with the combinatorial expression profiles in the given mode is obtained, then order different combinations by the mean values of the local clustering coefficients. We use the latter in this work. As it is biologically more reasonable that the target gene should be simultaneously activated or delayed in its expression accumulation compared with the regulators, we have also set this restrictive condition in the algorithm.

### Expression data and transcription network

As previously reported [15], we used the high-resolution (short interval) time-series microarray data (17 time points with uniform interval of 10 minutes) originally generated for yeast cell-cycle analysis with whole-genome yeast oligonucleotide chips that included over 6,000 open reading frames [36]. After removal of all the negative expression levels in the scaled measurements and all the dubious genes and the genes now deleted in the *Saccharomyces *Genome Database [38], 5,680 genes were included in our analysis. We directly utilized the original expression values normalized by Cho *et al*. [36], rather than the log-transformed values, for further calculation. With the same procedures, we included 5,138 genes from the Spellman time-series dataset [34]. To make them comparable, we extracted the data with a 170 minute measurement (90-250 minutes, cdc15-based data; the percentage of dumbbells reached 100% at 90 minutes in the work of Spellman *et al*. [34]). Note that not only those genes considered to be involved in cell-cycle process were included, because many other biological processes or pathways may also be regulated in the synchronized yeast cell cultures. From the study of transcriptional response of steady-state yeast cultures with a low-level (0.2 g/l) glucose pulse perturbation [44], we chose 5 time points from 2 to 10 minutes (with an interval of 2 minutes). We included 6,656 probe sets in this glucose perturbation data. We also included 6,049 probe sets from the data originally used for the analysis of expression in yeast cells in response to constant 0.32 mM H_2_O_2 _stress [45]. We also chose 5 time points from 10 to 50 minutes (with an interval of 10 minutes; most values are missed at 60 minutes).

The yeast transcriptional regulatory network used was previously established [20] by integrating results of genetic [37], biochemical [64], and ChIP-chip experiments [65]. In the established network [20], there were 7,074 interactions and 1,110 FFLs. From the Cho dataset of this work, we included 6,105 of these interactions after projecting the 5,680 genes and removing the auto-regulatory interactions. For a given gene, providing that only two (or only three) regulators are known to control this target gene, and providing that the expression profiles of the two- (or three-) regulator genes also exist in the dataset, the two- (or three-) regulator-to-one-target-gene network structure motif is identified from the regulatory network. Note that according to this definition, two-regulator-to-one-target-gene motifs are not subsets of three- or more regulator motifs.

Through this approach, we obtained 745 two- and 331 three-regulator motifs from the Cho dataset. In order to constrain the time-shift and the conversion efficiency of a given regulator to the same value among different target genes in a given convergence mode, we chose only the convergence modes in which the two or three regulators have more than one target gene. Therefore, 544 two- and 161 three-regulator motifs were eventually included from the Cho dataset. By the same approach, we obtained 219 two- and 38 three-regulator network structure motifs from the Spellman dataset. From the glucose pulse dataset, 557 two- and 168 three-regulator network motifs were included. We obtained 453 two- and 120 three-regulator motifs from the data of the H_2_O_2 _stress study.

### Statistical analysis

The *P *value of the LC coefficient between the expressions of a single regulator and its target gene presented in this work is identical to that between two genes at the genome scale. Consequently, the *P *value distribution table (Additional data file 8) can be directly obtained from previously published works [15,17] in which the correlation score has been calculated between two genes of a pair based on randomly shuffled data between different time points of the original data. In this work, for multi-regulator motifs, the LC coefficient was calculated between the combinatorial profile of the regulators and the expression profile of the target gene, that is, the LC coefficient is eventually calculated between expression profiles of two genes.

### Multiple hypothesis testing

In biology, a high correlation coefficient represents a high chance of having a good correlation, for example, between the expression of the studied genes or proteins. However, when the LC coefficient is directly compared among the single regulator, two-regulator, and three-regulator transcription network structure motifs, the relationship might not be rigorous, since, strictly speaking, the significance of a single variable model cannot be compared directly with multi-variable models in mathematics. In any specific convergence mode, there are a large number of possible combinations of time-shifts and conversion efficiencies. We thus asked whether similar results would be obtained by chance in randomly shuffled data.

To determine whether the distribution of success percentages at different thresholds is different for the original data and randomized data, we calculated the correlation scores of the two- and three-regulator motifs in randomly shuffled expression data and have subsequently performed both the one-tail paired Student's *t*-test and the Wilcoxon matched-pairs signed-ranks test. For easy comparison, all significant correlations in the cell-cycle datasets of this work are based on the LC coefficient threshold of 13, corresponding to a *P *value of 2.7E-3 between two genes of a pair. The range of LC coefficients between expression profiles with 17 time points is from 0-17 (Additional data file 8). The LC coefficients between expression profiles with 5 time points are distributed from 0-5 (Additional data file 8). In these data, a threshold of 4.93 corresponds to a *P *value of 2.7E-3 between two genes of a pair.

We also generated random networks by randomly choosing genes as regulators and target genes. The random networks are generated by keeping the same structure for each convergence mode in the original network and keeping the expression data intact. Keeping the same structure of the convergence modes is to make sure the random networks are comparable with the real network. In this way, the statistical results are more reliable since we need to constrain the time-shift and the conversion efficiency of a given regulator to the same value for different target genes in a given convergence mode.

Comparisons of success frequencies between the FFL and non-FFL groups have been performed by Yates chi-square statistics. However, the chi-square test is not suitable when the 'expected values' in any of the cells of the contingency table are below 10. In these cases, a two-tail Fisher's exact test has been employed.

To determine whether a given number of convergence modes have a contiguous short concentrative distribution area (time points or conversion efficiencies) among a certain total number of modes can be obtained by chance, we used the binomial distribution test. Of note, the possibility of a contiguous distribution was also calculated. The final *P *values in these cases are the product of the *P *value of the binomial distribution test and the possibility to obtain a contiguous distribution.

### False discovery rate

The FDR is a more direct measure of the overall accuracy of a set of significant features. To estimate the accuracy of identifying a significant correlation in the two-regulator and three-regulator motifs, we also performed an FDR calculation at given thresholds. The estimation of FDR follows the algorithm proposed by Storey and Tibshirani [66]. The FDR at a given threshold can be calculated by:

$$FDR_{th}=\frac{\pi_{0}\times m\times th}{num\_success\_th}$$

where *π*_0 _is the proportion of features that are truly null (*π*_0 _is calculated by the method as described elsewhere [66]); *m *represents the whole number of the two- or three-regulator motifs; *th *represents the success percentage at the given threshold obtained in random expression data or in random networks; and *num_success_th *is the number of the motifs that succeed at the given threshold. Additional information related to FDR is provided in Additional data file 8.

## Abbreviations

FDR = false discovery rate; FFL, feed-forward loop; LC, local clustering coefficient; PCC, Pearson correlation coefficient; TC, trend correlation method.

## Authors' contributions

F.H. designed the study, performed the experiments and the algorithm development, and drafted the manuscript. R.B. supervised the study, revised the manuscript and improved the work design. A.P.Z. and J.B. improved the work design.

## Additional data files

The following additional data are available with the online version of this paper. Additional data file 1 is a table listing detailed results of all the two-regulator motifs in the Cho data and the distribution of the time-shift and conversion efficiencies of given regulators among different convergence modes. Additional data file 2 is a table listing detailed results of all the three-regulator motifs in the Cho data. Additional data file 3 is a table listing detailed results of all the two-regulator motifs in the Spellman data and comparison with the results from the Cho data. Additional data file 4 is a table listing detailed results of all the three-regulator motifs in the Spellman data and comparison with the results from the Cho data. Additional data file 5 is a table listing detailed results of all the two- and three-regulator motifs in the glucose pulse perturbation data. Additional data file 6 is a table listing detailed results of all the two- and three-regulator motifs in the H_2_O_2 _stress data. Additional data file 7 includes a summary and references for genes in the present work involved in the cell-cycle process as annotated by the Gene Ontology database. Additional data file 8 includes results for convergence modes from the randomized expression data with 17 time points, for random networks, and details of FDRs, as well as the number of combinations in all the cases listed in Table 1, and the *P *value distribution figures for time-series datasets with 17 time points and 5 time points.

## Supplementary Material

Additional data file 1 Detailed results of all the two-regulator motifs in the Cho data and the distribution of the time-shift and conversion efficiencies of given regulators among different convergence modes. Click here for file

Additional data file 2 Detailed results of all the three-regulator motifs in the Cho data. Click here for file

Additional data file 3 Detailed results of all the two-regulator motifs in the Spellman data and comparison with the results from the Cho data. Click here for file

Additional data file 4 Detailed results of all the three-regulator motifs in the Spellman data and comparison with the results from the Cho data. Click here for file

Additional data file 5 Detailed results of all the two- and three-regulator motifs in the glucose pulse perturbation data. Click here for file

Additional data file 6 Detailed results of all the two- and three-regulator motifs in the H_2_O_2 _stress data. Click here for file

Additional data file 7 Summary and references for genes in the present work involved in the cell-cycle process as annotated by the Gene Ontology database. Click here for file

Additional data file 8 Results for convergence modes from the randomized expression data with 17 time points, for random networks, and details of FDRs, as well as the number of combinations in all the cases listed in Table 1, and the *P *value distribution figures for time-series datasets with 17 time points and 5 time points. Click here for file
