# Supplementary material for: Dynamic cumulative activity of transcription factors as a mechanism of quantitative gene regulation
Source: Genome Biol. 2007 Sep 4;8(9):R181. doi: 10.1186/gb-2007-8-9-r181 (PMC2375019; doi:10.1186/gb-2007-8-9-r181)
Supplement: Additional data file 7 — Summary and references for genes in the present work involved in the cell-cycle process as annotated by the Gene Ontology database. [file gb-2007-8-9-r181-S7.doc]

Supplementary File 1 for the manuscript by Feng He, Jan Buer, An-Ping Zeng and Rudi Balling (2007). Shifted cumulative activity of transcription factors as a mechanism of quantitative gene regulation.

References for cell cycle processes annotation (according to Gene Ontology (GO) ) used in this work.

| ACE2 | (McBride et al. 1999) | G1-specific transcription in mitotic cell cycle |
| --- | --- | --- |
| FAR1 | (Peter et al. 1993) | cell cycle arrest |
| FKH1 | (Zhu et al. 2000) | regulation of progression through cell cycle |
| FKH2 | (Zhu et al. 2000) | regulation of progression through cell cycle |
| FKH2 | (Pic et al. 2000) | G2-specific transcription in mitotic cell cycle |
| HCM1 | (Horak et al. 2002) | G1/S-specific transcription in mitotic cell cycle |
| MBP1 | (Koch et al. 1993) | regulation of progression through cell cycle |
| NDD1 | (Loy et al. 1999) | G2/M-specific transcription in mitotic cell cycle |
| PLM2 | (Horak et al. 2002) | G1/S-specific transcription in mitotic cell cycle |
| POG1 | (Horak et al. 2002) | G1/S-specific transcription in mitotic cell cycle |
| POG1 | (Leza and Elion 1999) | re-entry into mitotic cell cycle after pheromone arrest |
| PPH22 | (Munoz et al. 2003) | G1/S transition of mitotic cell cycle |
| RPN4 | (Xie and Varshavsky 2001) | G2/M transition of mitotic cell cycle |
| SAP4 | (Luke et al. 1996) | G1/S transition of mitotic cell cycle |
| SWI4 | (Munoz et al. 2003) | G1/S transition of mitotic cell cycle |
| SWI4 | (Koch and Nasmyth 1994) | cell cycle |
| SWI5 | (McBride et al. 1999) | G1-specific transcription in mitotic cell cycle |
| SWI6 | (Leem et al. 1998) | G1/S-specific transcription in mitotic cell cycle |
| SYF2 | (Ben-Yehuda et al. 2000) | cell cycle |
| TOS4 | (Horak et al. 2002) | G1/S-specific transcription in mitotic cell cycle |
| TOS8 | (Horak et al. 2002) | G1/S-specific transcription in mitotic cell cycle |
| TYE7 | (Horak et al. 2002) | G1/S-specific transcription in mitotic cell cycle |
| YAP5 | (Horak et al. 2002) | G1/S-specific transcription in mitotic cell cycle |
| YHP1 | (Horak et al. 2002) | G1/S-specific transcription in mitotic cell cycle |
| YHP1 | (Pramila et al. 2002) | regulation of progression through mitotic cell cycle |
| YOX1 | (Horak et al. 2002) | G1/S-specific transcription in mitotic cell cycle |
| YOX1 | (Pramila et al. 2002) | regulation of progression through mitotic cell cycle |
| ZPR1 | (Gangwani et al. 1998) | regulation of progression through cell cycle |

References

Ben-Yehuda, S., Dix, I., Russell, C.S., McGarvey, M., Beggs, J.D., and Kupiec, M. 2000. Genetic and physical interactions between factors involved in both cell cycle progression and pre-mRNA splicing in Saccharomyces cerevisiae. *Genetics* **156:** 1503-1517.

Gangwani, L., Mikrut, M., Galcheva-Gargova, Z., and Davis, R.J. 1998. Interaction of ZPR1 with translation elongation factor-1alpha in proliferating cells. *J Cell Biol* **143:** 1471-1484.

Horak, C.E., Luscombe, N.M., Qian, J., Bertone, P., Piccirrillo, S., Gerstein, M., and Snyder, M. 2002. Complex transcriptional circuitry at the G1/S transition in Saccharomyces cerevisiae. *Genes Dev* **16:** 3017-3033.

Koch, C., Moll, T., Neuberg, M., Ahorn, H., and Nasmyth, K. 1993. A role for the transcription factors Mbp1 and Swi4 in progression from G1 to S phase. *Science* **261:** 1551-1557.

Koch, C. and Nasmyth, K. 1994. Cell cycle regulated transcription in yeast. *Curr Opin Cell Biol* **6:** 451-459.

Leem, S.H., Chung, C.N., Sunwoo, Y., and Araki, H. 1998. Meiotic role of SWI6 in Saccharomyces cerevisiae. *Nucleic Acids Res* **26:** 3154-3158.

Leza, M.A. and Elion, E.A. 1999. POG1, a novel yeast gene, promotes recovery from pheromone arrest via the G1 cyclin CLN2. *Genetics* **151:** 531-543.

Loy, C.J., Lydall, D., and Surana, U. 1999. NDD1, a high-dosage suppressor of cdc28-1N, is essential for expression of a subset of late-S-phase-specific genes in Saccharomyces cerevisiae. *Mol Cell Biol* **19:** 3312-3327.

Luke, M.M., Della Seta, F., Di Como, C.J., Sugimoto, H., Kobayashi, R., and Arndt, K.T. 1996. The SAP, a new family of proteins, associate and function positively with the SIT4 phosphatase. *Mol Cell Biol* **16:** 2744-2755.

McBride, H.J., Yu, Y., and Stillman, D.J. 1999. Distinct regions of the Swi5 and Ace2 transcription factors are required for specific gene activation. *J Biol Chem* **274:** 21029-21036.

Munoz, I., Simon, E., Casals, N., Clotet, J., and Arino, J. 2003. Identification of multicopy suppressors of cell cycle arrest at the G1-S transition in Saccharomyces cerevisiae. *Yeast* **20:** 157-169.

Peter, M., Gartner, A., Horecka, J., Ammerer, G., and Herskowitz, I. 1993. FAR1 links the signal transduction pathway to the cell cycle machinery in yeast. *Cell* **73:** 747-760.

Pic, A., Lim, F.L., Ross, S.J., Veal, E.A., Johnson, A.L., Sultan, M.R., West, A.G., Johnston, L.H., Sharrocks, A.D., and Morgan, B.A. 2000. The forkhead protein Fkh2 is a component of the yeast cell cycle transcription factor SFF. *Embo J* **19:** 3750-3761.

Pramila, T., Miles, S., GuhaThakurta, D., Jemiolo, D., and Breeden, L.L. 2002. Conserved homeodomain proteins interact with MADS box protein Mcm1 to restrict ECB-dependent transcription to the M/G1 phase of the cell cycle. *Genes Dev* **16:** 3034-3045.

Xie, Y. and Varshavsky, A. 2001. RPN4 is a ligand, substrate, and transcriptional regulator of the 26S proteasome: a negative feedback circuit. *Proc Natl Acad Sci U S A* **98:** 3056-3061.

Zhu, G., Spellman, P.T., Volpe, T., Brown, P.O., Botstein, D., Davis, T.N., and Futcher, B. 2000. Two yeast forkhead genes regulate the cell cycle and pseudohyphal growth. *Nature* **406:** 90-94.
