# Supplementary material for: Dynamic cumulative activity of transcription factors as a mechanism of quantitative gene regulation
Source: Genome Biol. 2007 Sep 4;8(9):R181. doi: 10.1186/gb-2007-8-9-r181 (PMC2375019; doi:10.1186/gb-2007-8-9-r181)
Supplement: Additional data file 8 — Results for convergence modes from the randomized expression data with 17 time points, for random networks, and details of FDRs, as well as the number of combinations in all the cases listed in Table 1, and the P value distribution figures for time-series datasets with 17 time points and 5 time points. [file gb-2007-8-9-r181-S8.doc]

Supplementary File 2 for the manuscript by Feng He, Jan Buer, An-Ping Zeng and Rudi Balling (2007). Shifted cumulative activity of transcription factors as a mechanism of quantitative gene regulation.

List of materials in this supplementary file.

Table S7. Success percentages at different thresholds in the two-regulator motifs from the real Cho expression data and randomized (shuffled between different time points) Cho data.

Table S8 Success percentages at different thresholds in the three-regulator motifs from the real Cho expression data and randomized (shuffled between different time points) Cho data.

Table S9 Success percentages at different thresholds in the two-regulator motifs from the real transcription network and the randomly generated transcription networks.

Table S10 Success percentages at different thresholds in the three-regulator motifs from the real transcription network and the randomly generated transcription networks.

Table S11. Number of combinations in different cases listed in Table 1 by considering the number of possible time shifts and conversion efficiencies.

Figure S1. *P*-value distribution of local clustering coefficient in 17 time-point datasets between two genes of pairs from the distribution table generated in the work (He and Zeng, 2006).

Figure S2. Distribution of local clustering coefficient between two genes of pairs from the time-series data with 5 time points.

Table S7. Success percentages at different thresholds in the two-regulator motifs from the real Cho expression data and randomized (shuffled between different time points) Cho data.

| Local clustering coefficient | *P*-value* threshold  Between two genes | Success percentage  at Cho data | Success percentage  at random data |
| --- | --- | --- | --- |
| 10 | 0.152 | 64.15% | 43.26% |
| 10.5 | 9.54E-2 | 56.99% | 35.42% |
| 11 | 5.74E-2 | 52.39% | 27.14% |
| 11.5 | 3.32E-2 | 47.24% | 21.63% |
| 12 | 1.85E-2 | 39.52% | 17.40% |
| 12.5 | 9.81E-3 | 35.85% | 13.36% |
| **13** | **2.70E-3** | **32.90%** | **11.95%** |
| 13.5 | 2.28E-3 | 17.46% | 3.80% |
| 14 | 9.39E-4 | 12.32% | 1.29% |
| 14.5 | 3.29E-4 | 6.43% | 0.61% |
| 15 | 8.85E-5 | 2.39% | 0.25% |
| 15.5 | 1.56E-5 | 1.10% | 0.00% |

*the *P*-value is calculated based on the local clustering coefficient between two genes of pairs (**see Fig. S1**) rather than two-regulator motifs. It is used to show how high the local clustering coefficient is.

The paired Student *t* test rejects the null hypothesis that the mean of success percentages at different thresholds in the real expression data is less than or equal to those in the random data with a significance level 3.35E-5 for the two-regulator motifs.

Because we don’t know whether the distributional assumption of the normal-theory-based *t* tests is satisfied in the distribution of the success percentage, we have also used the nonparametric test Wilcoxon matched-pairs signed-ranks test ( *P*<4.88E-4).

According to the algorithm proposed by Storey and Tibshirani (2003), π_0_ is 0.465. The value is estimated by function sm.spline of R package with spar 0.001. The False discovery rate (FDR) at the threshold 13 is 0.168. The P-value used for FDR calculation is from success percentages at random data rather than the *P*-value in the second column in the above table.

Table S8 Success percentages at different thresholds in the three-regulator motifs from the real Cho expression data and randomized (shuffled between different time points) Cho data.

| Local clustering coefficient | *P*-value* threshold between two genes | Success percentage  at Cho data | Success percentage  at random data |
| --- | --- | --- | --- |
| 10 | 0.152 | 90.68% | 73.91% |
| 10.5 | 9.54E-2 | 88.82% | 68.53% |
| 11 | 5.74E-2 | 83.85% | 63.77% |
| 11.5 | 3.32E-2 | 76.40% | 58.18% |
| 12 | 1.85E-2 | 73.29% | 52.80% |
| 12.5 | 9.81E-3 | 69.57% | 47.83% |
| 13 | 2.70E-3 | 67.08% | 47.20% |
| 13.5 | 2.28E-3 | 50.31% | 24.64% |
| 14 | 9.39E-4 | 36.02% | 13.66% |
| 14.5 | 3.29E-4 | 21.12% | 7.25% |
| 15 | 8.85E-5 | 9.32% | 2.69% |
| 15.5 | 1.56E-5 | 3.73% | 0.83% |

*the *P*-value is calculated based on the local clustering coefficient between two genes of pairs (**see Fig. S1**) rather than three-regulator motifs. It is used to show how high the local clustering coefficient is.

The paired Student *t* test rejects the null hypothesis that the mean of success percentages at different thresholds in the real expression data is less than or equal to those in the random data with a significance level 9.4E-7 in the three-regulator motifs.

Because we don’t know whether the distributional assumption of the normal-theory-based *t* tests is satisfied in the distribution of the success percentage, we also have used the nonparametric test Wilcoxon matched-pairs signed-ranks test ( *P*<4.88E-4).

For the three-regulator motif, π_0_ is 0.176.

The false discovery rate at threshold 13 is 0.124.

Table S9 Success percentages at different thresholds in the two-regulator motifs from the real transcription network and the randomly generated transcription networks.

| Local clustering coefficient | *P*-value* threshold  Between two genes | Success percentage  at Cho data | Success percentage  at random data |
| --- | --- | --- | --- |
| 10 | 0.152 | 64.15% | 57.31% |
| 10.5 | 9.54E-2 | 56.99% | 50.41% |
| 11 | 5.74E-2 | 52.39% | 43.80% |
| 11.5 | 3.32E-2 | 47.24% | 38.47% |
| 12 | 1.85E-2 | 39.52% | 32.86% |
| 12.5 | 9.81E-3 | 35.85% | 29.60% |
| 13 | 2.70E-3 | 32.90% | 27.30% |
| 13.5 | 2.28E-3 | 17.46% | 12.55% |
| 14 | 9.39E-4 | 12.32% | 6.57% |
| 14.5 | 3.29E-4 | 6.43% | 3.13% |
| 15 | 8.85E-5 | 2.39% | 1.06% |
| 15.5 | 1.56E-5 | 1.10% | 0.46% |

We have also generated random networks by randomly choosing genes as regulators and target genes but meanwhile keeping the same structure for each convergence mode and keeping the expression data intact. The purpose of keeping the same structure of the convergence mode is to make sure the random networks are more comparable and the statistical results are more reliable since we need to constrain the time-shift and the conversion efficiency of the same regulator to the same for different target genes in the same convergence mode.

One-tail paired Student’s *t* test: *P*=6.81E-6; Wilcoxon matched-pairs signed-ranks test: *P*=4.88E-4.

If one compares the real transcription network with the randomly generated transcription network,

π_0_ is 0.467 and the false discovery rate at threshold 13 is 0.338.

Table S10 Success percentages at different thresholds in the three-regulator motifs from the real transcription network and the randomly generated transcription networks.

| Local clustering coefficient | *P*-value* threshold between two genes | Success percentage  at Cho data | Success percentage  at random data |
| --- | --- | --- | --- |
| 10 | 0.152 | 90.68% | 83.85% |
| 10.5 | 9.54E-2 | 88.82% | 79.09% |
| 11 | 5.74E-2 | 83.85% | 73.29% |
| 11.5 | 3.32E-2 | 76.40% | 66.46% |
| 12 | 1.85E-2 | 73.29% | 61.70% |
| 12.5 | 9.81E-3 | 69.57% | 57.35% |
| 13 | 2.70E-3 | 67.08% | 55.90% |
| 13.5 | 2.28E-3 | 50.31% | 35.61% |
| 14 | 9.39E-4 | 36.02% | 21.33% |
| 14.5 | 3.29E-4 | 21.12% | 12.01% |
| 15 | 8.85E-5 | 9.32% | 7.45% |
| 15.5 | 1.56E-5 | 3.73% | 2.48% |

One-tail paired Student’s *t* test: 5.15E-6; Wilcoxon matched-pairs signed-ranks test: 4.88E-4.

If one compares the real transcription network with the randomly generated transcription network,

π_0_ is 0.468 and the false discovery rate at threshold 13 is 0.390.

Table S11. Number of combinations in different cases listed in Table 1 by considering the number of possible time shifts and conversion efficiencies.

|  | Before considering  multi-regulators | Two-regulator motifs | | | Three-regulator motifs | |
| --- | --- | --- | --- | --- | --- | --- |
| Possible time delays from regulator(s) to target genes | + | + | + | + | + | + |
| Conversion efficiency (non-  negative) |  | + | + | + | + | + |
| Possible opposite regulation between regulators |  |  | + | + | + | + |
| Possible time delays among regulators |  |  |  | + |  | + |
| Whole number trials | 289* | 289*11*11  =34,969 | 289*21*21  =127,449 | 289*21*21*10*10  =12744900 | 289*21*21*21  =2,676,429 | 289*21*21*21  *1000=2.6764E9 |

+ indicate the corresponding factor is considered in the corresponding case.

* This number indicates all the possible local similarities between two expression profiles. The time shift from time point 14 in one gene to time point 15 in another gene is equal to that from time point 15 in one gene to time point 16 in another gene in terms of the number of time shift. However, the ranges of maximal local alignment are different. For details, see the reference (Qian et al., 2001) and/or related references (He and Zeng, 2006)

In the current study, we have constrained the maximal time-shift between regulators to one cell cycle (10 time points) for the data with 17 time points.

Figure S1. *P*-value distribution of local clustering coefficient in 17 time-point datasets between two genes of pairs from the distribution table generated in the work (He and Zeng, 2006).

Figure S2. Distribution of local clustering coefficient between two genes of pairs from the time-series data with 5 time points. For the random data, P is the traditional *P*-value. For the real data, P is the percentage of gene pairs, which are higher than the corresponding local clustering coefficient.

From this figure, we can see that the frequency of gene pairs in the original data and random data with 5 time points is quite difficult to be distinguished.
